# Design and operation of an autosampler controlled flow-injection preconcentration system for lead determination by flame atomic absorption spectrometry

**DOI:** 10.1155/S1463924689000088

**Published:** 1989

**Authors:** S. R. Bysouth, J. F. Tyson, P. B. Stockwell

**Affiliations:** 1 Chemistry Department University of Technology Leicestershire Loughborough LE11 3TU UK; 2 P.S. Analytical Ltd Arthur House, Unit B4 Chaucer Business Park Watery Lane, Kemsing, Kent Sevenoaks TN15 6QY UK

## Abstract

Flow-injection manifolds are described which allow the preconcentration of lead for flame atomic absorption determinations, using columns contained within the sample loop of an injection valve. An interface was designed which allowed the valves and pump in the system to be controlled by an autosampler which enabled precise timing of preconcentration and elution steps. The effects of sample flow rate, buffer pH and buffer type for preconcentration and eluent concentration and flow rate were investigated in order to obtain optimum performance of the system. A 50-times improvement in detection limits over conventional sample introduction was obtained for a sample volume of approximately 12 ml, preconcentrated for 150 s. The injection of eluent, as opposed to the use of a continuously flowing eluent stream, enabled this reagent to be conserved.


Journal of Automatic Chemistry Vol. 11, No. 1 (January–February 1989), pp. 36–39

### Design and operation of an autosampler controlled flow-injection preconcentration system for lead determination by flame atomic absorption spectrometry

**S. R. Bysouth, J. F. Tyson**

*Chemistry Department, University of Technology, Loughborough, Leicestershire LE11 3TU, UK*

**and P. B. Stockwell**

*P.S. Analytical Ltd, Arthur House, Unit B4, Chaucer Business Park, Watery Lane, Kemsing, Sevenoaks, Kent TN15 6QY, UK*

*Flow-injection manifolds are described which allow the preconcentration of lead for flame atomic absorption determinations, using columns contained within the sample loop of an injection valve. An interface was designed which allowed the valves and pump in the system to be controlled by an autosampler which enabled precise timing of preconcentration and elution steps. The effects of sample flow rate, buffer pH and buffer type for preconcentration and eluent concentration and flow rate were investigated in order to obtain optimum performance of the system. A 50-times improvement in detection limits over conventional sample introduction was obtained for a sample volume of approximately 12 ml, preconcentrated for 150 s. The injection of eluent, as opposed to the use of a continuously flowing eluent stream, enabled this reagent to be conserved.*

### Introduction

A number of applications of flow-injection techniques have been made to flame atomic absorption spectrometry [1]. Although manifolds can be connected directly to the nebulizer, the response of the spectrometer is dependent on the flow rate of sample into the nebulizer [2], and some adjustment to the manifold may be required. The optimum flow rate for maximum response when the sample enters the nebulizer as a discrete sample plug can be different from that found for analysis of a continuous sample stream.

Several batch methods of preconcentration have been developed including solvent extraction, precipitation, immobilization and electrodeposition. Most of these have been adapted to the flow-injection format for which retention on a small column of immobilized reagent appears most attractive, due to its simplicity. The manifolds which have been previously described operate using the injection of a large sample volume either by timed flow switching (timed injection) [3,4] or by using a large sample loop in an injection valve [5–7]. This second option does not allow the sample size to be varied without changing the size of the sample loop, unless multiple injections are made. In theory, timed injection should allow the sample volume to be infinitely varied. When the column is placed just before the nebulizer [5–7] the sample matrix will pass from the column to the nebulizer. This could cause nebulizer blockage or an unstable base-line. Furthermore, if the optimum flow rate for preconcentration is different from the optimum flow-injection nebulization flow rate, the flow rate must be changed during the analysis part of the cycle or a non-optimum or compromise flow rate must be used for either preconcentration or elution. By diverting the column effluent away from the nebulizer [4 and 7] these problems can be eliminated.

In this paper, the design and operation of a simple manifold for the preconcentration of lead is described.

### Experimental

*Preconcentration manifold design*

The preconcentration columns consisted of 40 mm lengths of glass tubing, of 2·5 mm i.d., packed with solid reagent. By placing the column within the sample loop of a rotary injection valve as shown in figure 1, the column could be switched out of the carrier stream to allow preconcentration to be performed at a flow rate different from that used for elution by the carrier.

The autosampler used (PS Analytical, 20.080) has three positions for the sample probe: in the wash-pot, in the sample vial and between the wash-pot and the sample vial. The sampling and wash times can be programmed individually using an integral keyboard and the probe position is indicated by LEDs for these two positions. An

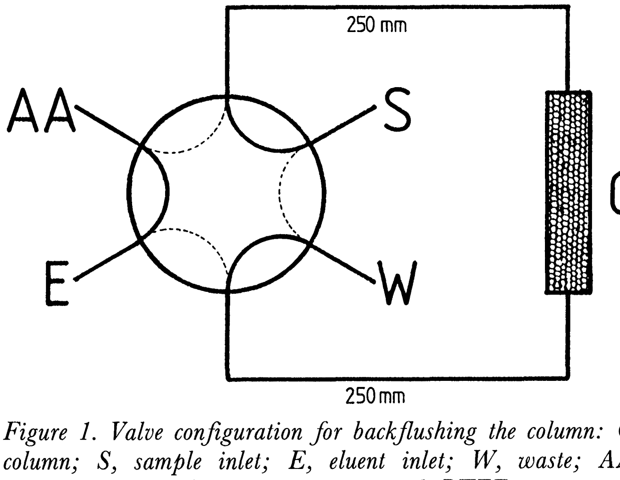

*Figure 1. Valve configuration for backflushing the column: C, column; S, sample inlet; E, eluent inlet; W, waste; AA, spectrophotometer. Tubing was 0·5 mm i.d. PTFE.*

0142–0453/89 $3.00 © 1989 Taylor & Francis Ltd.

*Table 1. Interface signals available on the autosampler.*

| Pin number | Name | Description |
|---|---|---|
| 1 and 9 | Ground | 0 V |
| 6 and 8 | Power | +5 V |
| 2 | A | Control logic inputs |
| 10 | B | Control logic inputs |
| 11 | C | Control logic inputs |
| 12 | D | Control logic inputs |
| 13 | Washed timed | Short 3·5 V pulse before sampling |
| 14 | Count | Short 5 V pulse for every sample position passed during rotation of the turntable |
| 15 | Wash | 2 V level held high when the probe is in the wash-pot |
| 7 | Sample | 2 V level held high when the probe is in the sample vial. |

interface socket is provided at the rear of the autosampler and the signals available are given in table 1. A simple circuit (shown in figure 2) was constructed which modified the output from the interface to allow two valves (PS Analytical, T-series) to be switched in tandem and a peristaltic pump (LKB/Pharmacia, Microperpex 2132) to be stopped when the sample probe was between the sample vial and the wash-pot. The complete timing sequence is given in figure 3.

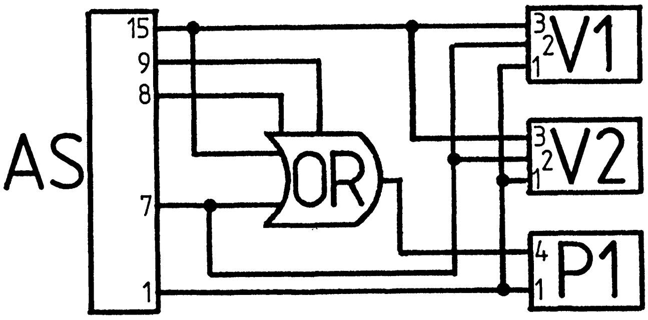

*Figure 2. Valve and pump interface circuit: AS, autosampler socket; OR, Or gate SN74LS32N; for other symbols see text.*

| AS PROBE | WASH | MOVE | SAMPLE | MOVE |
|---|---|---|---|---|
| V1,V2 | INJECT | | RETURN | |
| P1 | RUN | STOP | RUN | STOP |

time ⟶

*Figure 3. Timing sequence for the autosampler AS, valves V1, V2 and pump P1.*

Initially, a single-valve manifold was constructed, as shown in figure 4. In this manifold, the sample and buffer are merged by pumping via pump P1, before passing to the column where the lead is retained. After the appropriate time interval the probe leaves the sample vial which stops pump P1. When the probe enters the wash-pot, pump P1 is restarted and valve V1 switches to the inject position. The eluent stream which is continuously pumped by pump P2 (Ismatec, Mini-S 840), then

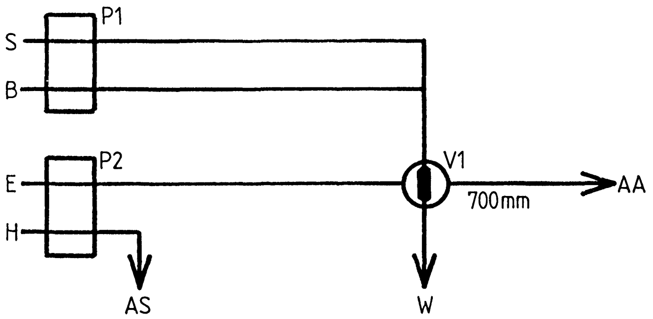

*Figure 4. Manifold 1: B, buffer; H, water. For other symbols see text.*

back-flushes the lead from the column to the spectrometer. Meanwhile, any sample solution remaining in the probe, pump P1 and associated tubing is washed to waste by a combination of wash solution and buffer. This manifold was used for investigation of optimum preconcentration flow rate, optimum buffer pH and eluent concentration.

A second manifold was constructed (shown in figure 5), which enabled the simultaneous injection of a 79 µl slug of eluent via valve V2 when the column was switched in line. This enabled the eluent to be conserved during the preconcentration step. This manifold was used for investigations of detection limits. For all the experiments described, except during optimization of preconcentration flow rate, a sampling time of 150 s and a wash time of 40 s were used.

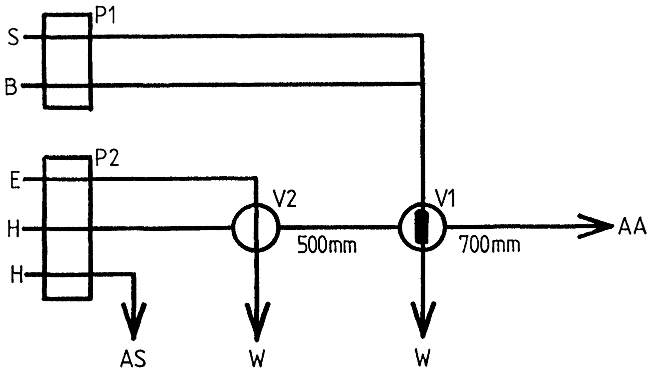

*Figure 5. Manifold 2: symbols as figure 4.*

Two other manifolds were used. One enabled a lead sample and buffer stream to be merged before entering a column which was directly connected to the nebulizer. This was used to investigate the effect of buffer type. The other consisted of an injection valve which allowed injection of a lead solution into a water carrier and transportation to the nebulizer, without preconcentration. This manifold was used to optimize the elution flow rate which, although independent of the preconcentration flow rate, is the same as the nebulizer flow rate.

Teflon tubing, 0·5 mm i.d. (Anachem) and low-pressure T-pieces (Anachem) were used for all manifold connections.

### Apparatus

The manifolds as described above were connected to a Philips Scientific SP9 flame atomic absorption spectrometer, which was optimized for the determination of lead. The response was recorded using a chart recorder (Philip AR55). All reagents were either SpectrosoL or AristaR grade (BDH Chemicals). Water was reagent grade obtained from a Liquipure R.G. reverse osmosis and deionization unit. To prevent the adsorption of lead from solutions, onto the walls of the glassware, one drop of nitric acid (SG 1·412) was included per 100 ml in all lead solutions.

### Procedures

*Optimization of preconcentration flow rate*

When the preconcentration manifolds shown in figures 4 and 5 are used, preconcentration is carried out at the combined buffer and sample flow rates. For the LKB Microperpex pump, two sizes of pump tubing can be supplied which allows flow ratios of 1 : 1 and 5 : 1 to be used. To minimize the dilution of the sample, a flow ratio of 5 : 1 sample to buffer was used. The buffer was a 1 M sodium acetate solution of pH 7·0. The flow rates and the corresponding preconcentration times were set so that the volume of a 100 ng ml$^{-1}$ lead solution that was preconcentrated was approximately 12 ml. The mass of sample used during each preconcentration cycle was measured in order that preconcentration from non-equivalent sample volumes could be taken into account.

*pH of preconcentration buffer*

A range of Universal buffer solutions [8] were made and sample solutions containing 0·2, 0·4 and 1·0 μg ml$^{-1}$ lead were merged with each buffer in turn, and preconcentrated at a sample flow rate of 4·9 ml min$^{-1}$.

*Effect of buffer type*

The column effluent was monitored when two buffers were merged with either a 10 μg ml$^{-1}$ lead solution or a blank solution. The two buffers consisted of 19 g l$^{-1}$ borax adjusted to pH 8 with either citric or boric acid. The column was eluted after each preconcentration step by flushing with 1 M hydrochloric acid solution.

*Eluent concentration*

A 1·0 μg ml$^{-1}$ lead solution was preconcentrated by merging with a borax/boric acid buffer (pH 8) and eluted using a stream of either 0·25, 0·5, 0·75 or 1 M hydrochloric acid.

*Eluent flow rate*

The optimum flow rate for the injection of a 10 μg ml$^{-1}$ lead solution into a single line manifold with a water carrier stream was found. This value was taken as the optimum eluent flow rate.

*Detection limits*

The original manifold was modified as described above to include an injection valve for the injection of eluent. Calibrations were generated from solutions containing 0, 10, 20 and 30 ng ml$^{-1}$ lead, five preconcentration and elution cycles being performed for each solution. Elution was performed by injecting 1 M hydrochloric acid. Detection limits were calculated from the resulting calibration curves [9].

### Results and discussion

The results obtained from the preconcentration of solutions at various flow rates are presented in table 2. These results show that the efficiency of preconcentration decreases by 3% when the sample flow rate is increased from 2·64 ml min$^{-1}$ to 4·86 ml min$^{-1}$. If the flow rate is increased to 7·20 ml min$^{-1}$, the efficiency drops by a further 10·5%. When analysis time is taken into account the efficiency of preconcentration becomes less important as its reduction is more than compensated for by the increase in sample volume from which more lead can be extracted in the same time. For subsequent experiments a sample flow rate of 4·86 ml min$^{-1}$ was used.

*Table 2. Effect of flow rate on preconcentration efficiency.*

| Measured sample flow rate (ml min$^{-1}$) | Total flow rate (ml min$^{-1}$) | Mass of sample aspirated (g) | Peak height for 100 μl$^{-1}$ (absorbance) | Peak height volume: efficiency (ml$^{-1}$) |
|---|---|---|---|---|
| 7·2 | 8·64 | 12·0 | 0·138 | 0·0115 |
| 4·86 | 5·83 | 12·2 | 0·158 | 0·0129 |
| 3·84 | 4·61 | 12·8 | 0·164 | 0·0128 |
| 3·18 | 3·82 | 13 2 | 0·175 | 0·0133 |
| 2·64 | 3·17 | 13·2 | 0·176 | 0·0133 |

The effect of changing the pH of the buffer used for preconcentration is shown in figure 6. For the universal buffers used, the species present do not change, only their concentration. Hence there will not be a great difference in any interference of the preconcentration process by constituents of the buffer at different pH values. The optimum pH appears to be at or above pH 8. At pH 8, the solubility product of lead hydroxide is not exceeded until the lead concentration is greater than 500 μg ml$^{-1}$. When the effluent of the column was monitored whilst different buffers were merged with a sample, the trace shown in figure 7 was obtained. The erratic signal shows that the borax/citric acid buffer is unsuitable for the preconcentration of lead in this system. It was hoped that such a

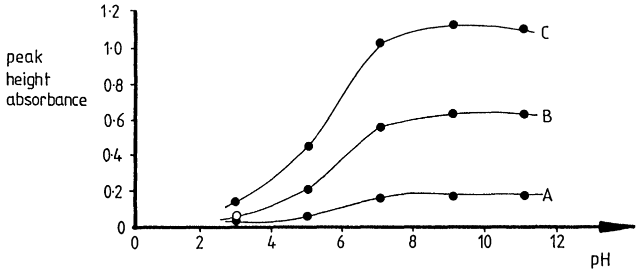

*Figure 6. Effect of pH on lead preconcentration: A, 0·2 μg ml$^{-1}$; B, 0·4 μg ml$^{-1}$; C, 1·0 μg ml$^{-1}$.*

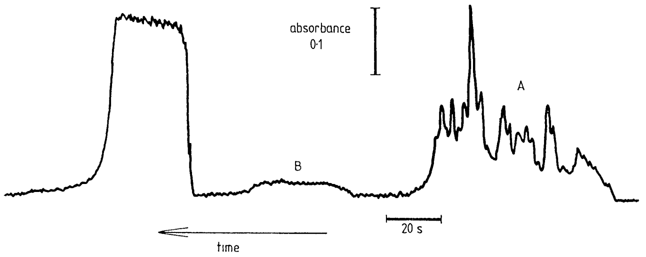

*Figure 7. Trace obtained whilst monitoring the effluent from the column. 10 μg ml$^{-1}$ lead merged with A: borax/citric acid, B: borax/boric acid buffers and C: water.*

buffer could be used to mask the competition for the immobilized reagent by other metals such as iron. The borax/boric acid buffer does not produce an erratic signal, and the signal is considerably less than that obtained without the column. This indicates that the buffer does not interfere with the efficient uptake of lead by the column.

When different acid concentrations were used to elute the column using a continuous flow of eluent, the peak heights were only reduced by 3·25%, when the acid was diluted from a 1 to 0·25 M solution. Thus, for the single valve manifold, eluent can be conserved by dilution. For the two valve manifold, injection of eluent and its subsequent transport through the manifold will cause eluent dilution. When this manifold was used for preconcentration, elution was performed using a large volume of 1 M acid, but the eluent is conserved as its flow rate into the injection valve is low.

Although elution is independent of the preconcentration flow rate, it is not independent of the nebulization flow rate. In these studies nebulizer flow rate was optimized at 5·3 ml min$^{-1}$ to give the maximum signal, rather than optimizing the elution flow rate. The peaks produced at this flow rate were smooth and sharp. Detection limits ranging from 2·8 ng ml$^{-1}$ to 1·4 ng ml$^{-1}$ were obtained which indicate that the system allows precise determinations of low lead concentrations with an increase in sensitivity of about 50 times. The detection limits can be further improved by increasing the preconcentration time at the expense of analysis time. Preliminary results show that peak heights are proportional to preconcentration time.

### Conclusion

Both systems performed well. By placing the column within the valve, a simple flow-injection manifold can be constructed which enables lead to be preconcentrated in a precise manner. The single valve manifold consumes a considerable volume of acid eluent but a low concentration of acid can be used to conserve reagent. If a large reservoir can be used, or recharging of the reservoir is not a problem, such a system is simpler and as effective as the system incorporating a second valve for elution by acid injection. Further additions could be made to these systems, such as a third valve for direct injection of samples and standards. In this way, samples with concentrations above the normal detection limit of the spectrometer can be analysed during the preconcentration of dilute lead samples, thus increasing the number of samples analyzed per hour.

### Acknowledgement

Financial support from the British Technology Group is gratefully acknowledged.

### References

1. Tyson, J. F., *Analyst*, **110** (1985), 419.
2. Tyson, J F., Adeeyinwo, C. E., Appleton, J. M. H., Bysouth, S. R., Idris, A. B. and Sarkissian, L. L., *Analyst*, **110** (1985), 487.
3. Malamas, F., Bengtsson, M. and Johansson, G., *Analytica Chimica Acta*, **160** (1984), 1.
4. Risinger, L., *Analytica Chimica Acta*, **179** (1986), 509.
5. Marshall, M. A. and Mottola, H. A., *Analytical Chemistry*, **57** (1985), 729.
6. Olsen, S., *Dansk Kemi*, **3** (1983), 68.
7. Olsen, S., Pessenda, L. C. R., Ruzicka, J. and Hansen, E. H., *Analyst*, **108** (1983), 905.
8. *Vogel's Textbook of Quantitative Inorganic Analysis*, Fourth Edition, Revised by Basset, J., Denny, R. C., Jeffery, G. H. and Mendham, J. (Longman, London and New York, 1978), p. 886.
9. Miller, J. C. and Miller, J. N., *Statistics for Analytical Chemistry* (Ellis Horwood, Chichester, 1984).

